# The world’s largest High Arctic lake responds rapidly to climate warming

**DOI:** 10.1038/s41467-018-03685-z

**Published:** 2018-03-29

**Authors:** Igor Lehnherr, Vincent L. St. Louis, Martin Sharp, Alex S. Gardner, John P. Smol, Sherry L. Schiff, Derek C. G. Muir, Colleen A. Mortimer, Neil Michelutti, Charles Tarnocai, Kyra A. St. Pierre, Craig A. Emmerton, Johan A. Wiklund, Günter Köck, Scott F. Lamoureux, Charles H. Talbot

**Affiliations:** 1 Department of Geography, University of Toronto-Mississauga, Mississauga, ON L5L 1C6 Canada; 2 Department of Biological Sciences, University of Alberta, Edmonton, AB T6G 2E9 Canada; 3 Department of Earth and Atmospheric Sciences, University of Alberta, Edmonton, AB T6G 2E3 Canada; 4 Jet Propulsion Laboratory, California Institute of Technology, Pasadena, CA 91109 USA; 5 Paleoecological Environmental Assessment and Research Lab, Department of Biology, Queen’s University, Kingston, ON K7L 3N6 Canada; 6 Department of Earth and Environmental Sciences, University of Waterloo, Waterloo, ON N2L 3G1 Canada; 7 Environment and Climate Change Canada, Canada Centre for Inland Waters, 867 Lakeshore Road, Burlington, ON L7S 1A1 Canada; 8 Agriculture and Agri-Food Canada, 1341 Baseline Road, Ottawa, ON K1A 0C5 Canada; 9 Institute for Interdisciplinary Mountain Studies (ÖAW-IGF), A-6020 Innsbruck, Austria; 10 Department of Geography and Planning, Queen’s University, Kingston, ON K7L 3N6 Canada

## Abstract

Using a whole-watershed approach and a combination of historical, contemporary, modeled and paleolimnological datasets, we show that the High Arctic’s largest lake by volume (Lake Hazen) has succumbed to climate warming with only a ~1 °C relative increase in summer air temperatures. This warming deepened the soil active layer and triggered large mass losses from the watershed’s glaciers, resulting in a ~10 times increase in delivery of glacial meltwaters, sediment, organic carbon and legacy contaminants to Lake Hazen, a >70% decrease in lake water residence time, and near certainty of summer ice-free conditions. Concomitantly, the community assemblage of diatom primary producers in the lake shifted dramatically with declining ice cover, from shoreline benthic to open-water planktonic species, and the physiological condition of the only fish species in the lake, Arctic Char, declined significantly. Collectively, these changes place Lake Hazen in a biogeochemical, limnological and ecological regime unprecedented within the past ~300 years.

## Abstract (web summary)

Arctic ecosystems are at threat due to the rapid nature of climate change and Arctic amplification. Here, the authors show that the watershed of Lake Hazen, the Arctic’s largest lake by volume, has undergone dramatic changes in response to as little as a ~1°C increase in summer air temperatures.

## Introduction

Arctic ecosystems are particularly sensitive to human-induced climate changes because of how rapidly they are warming due to Arctic amplification^1^. Climate model simulations, based on optimistic scenarios in which atmospheric carbon dioxide concentrations peak mid-century at ~550 ppm (RCP4.5), predict that summer near-surface air temperatures in the Canadian Arctic will increase by 3.2 °C by 2100, relative to 1986–2000 temperatures^2^. In the most northerly reaches of Canada on Northern Ellesmere Island, there has already been an ~1 °C increase in summer air temperatures during 2001–2012 relative to the 1986–2000 reference period^3^. These climate changes have potentially important consequences for Arctic ecosystems and, yet, there is a complete paucity of studies that document ecosystem-scale climate tipping points for inland watersheds. In fact, two recent syntheses emphasized the need for ecosystem-scale studies that integrate physical, chemical and ecological processes to better understand the climate–hydrology–biogeochemistry linkages that lead to ecosystem shifts^4,5^.

**Fig. 1:**
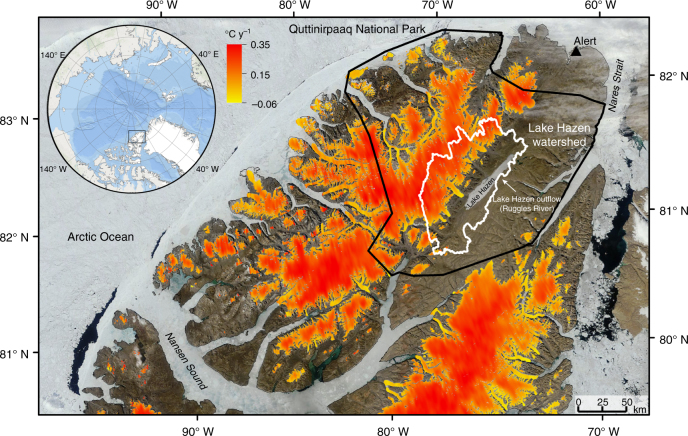
Location of the Lake Hazen watershed and changes in glacier surface temperatures. Changes in summer glacier surface temperatures (°C y^−1^) were quantified for the months of June, July and August for the period 2000–2012. The white line delineates the boundaries of the Lake Hazen watershed, and the glaciers within it (northern Ellesmere Island, Nunavut, Canada). The catchment area to lake area ratio for Lake Hazen is 12.7. The black line delineates the boundary of Quttinirpaaq National Park, Canada’s most northerly national park

Lake Hazen, located on northern Ellesmere Island (Nunavut, Canada; Fig. 1) is, by volume, the largest lake north of the Arctic Circle^6^. Arctic indigenous people of the Independence I Culture first arrived at Lake Hazen circa 2500 BC^7,8^. At various times since then, a succession of arctic-adapted cultures, including the modern Inuit, have hunted muskox in the region and fished Lake Hazen’s large population of Arctic Char (*Salvelinus alpinus*)^9^. The first people of European descent to explore the region were members of the ill-fated “Lady Franklin Bay Expedition” led by Lieutenant Adolphus Greely, as part of the USA contribution to scientific discovery during the first International Polar Year (1882–1883)^8^. Lake Hazen has a maximum depth of 267 m^10^, a surface area of 540 km^2^ and a catchment area of 6860 km^2^^11^. The NW half of its catchment is extensively glaciated, while the Hazen Plateau characterized by polar desert tundra lies to the SE. Lake Hazen is an ideal system for examining the impacts of recent climate change on Arctic freshwater ecosystems due to its large size, the variety of ecosystems found within its watershed (including glaciers, tundra, wetlands and other aquatic ecosystems), its location within protected Quttinirpaaq National Park and its history of scientific investigation, including “Operation Hazen” carried out during the International Geophysical Year of 1957–1958.

In this study, we investigate how a warming climate has impacted the Lake Hazen watershed from its glacier headwaters, all the way through to the Arctic Char at the top of the aquatic foodweb, using a combination of historical, contemporary, modeled and paleoliminological datasets. We hypothesized that, due to its large size and thermal inertia, Lake Hazen would be more resilient to Arctic warming (c.f. ref.^12^) than smaller aquatic ecosystems, a number of which have already undergone significant regime shifts^13–15^. However, we demonstrate that the Lake Hazen watershed was not resilient to even an ~1 °C relative increase in recent summer air temperatures. Accelerated melt in the cryosphere resulted in an ~10 times increase in delivery of glacial meltwaters, sediment, organic carbon and legacy contaminants to Lake Hazen and a reduction in summer lake ice cover. Changes to the physical and chemical components of the watershed caused an ecological reorganization of the algal (diatom) community assemblage and a decline in the physiological condition of Arctic Char.

## Results

### Watershed warming and declining lake ice cover

**Fig. 2:**
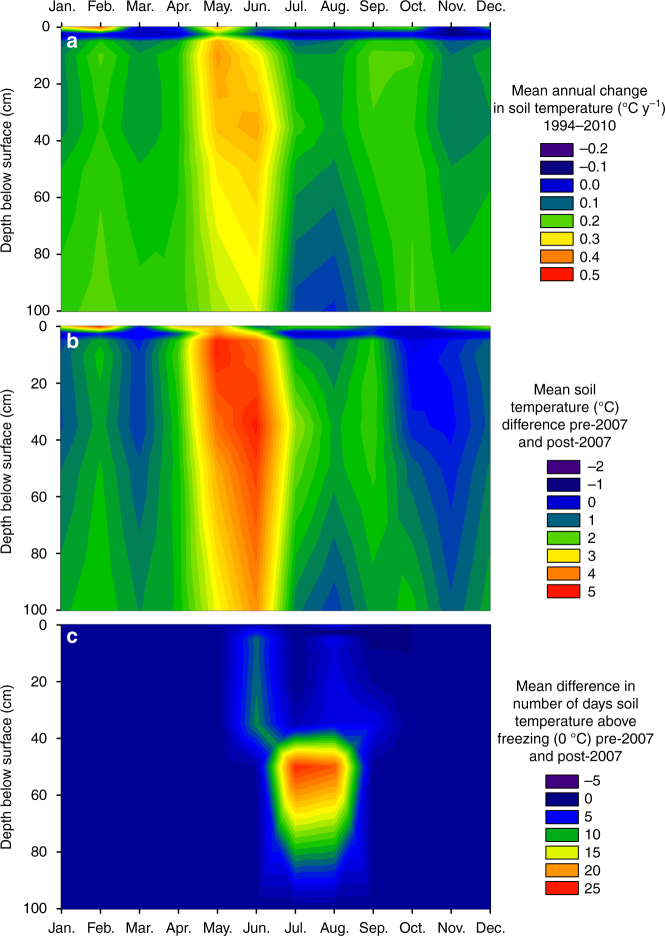
Changes in air and soil temperatures in the Lake Hazen watershed. **a** Mean annual change in monthly soil temperature (°C y^−1^) for the period 1994–2010. **b** Difference in mean monthly soil temperature (°C) between the periods of 2007–2010 and 1994–2006, indicating that soil temperatures have primarily increased since 2007. **c** Increase in the number of days mean daily soil temperature was above freezing during 2007–2010 compared with the 1994–2006 baseline, showing a recent thickening of the soil active layer. Depth 0 is shielded air temperature at 1 m above the soil surface

**Fig. 3:**
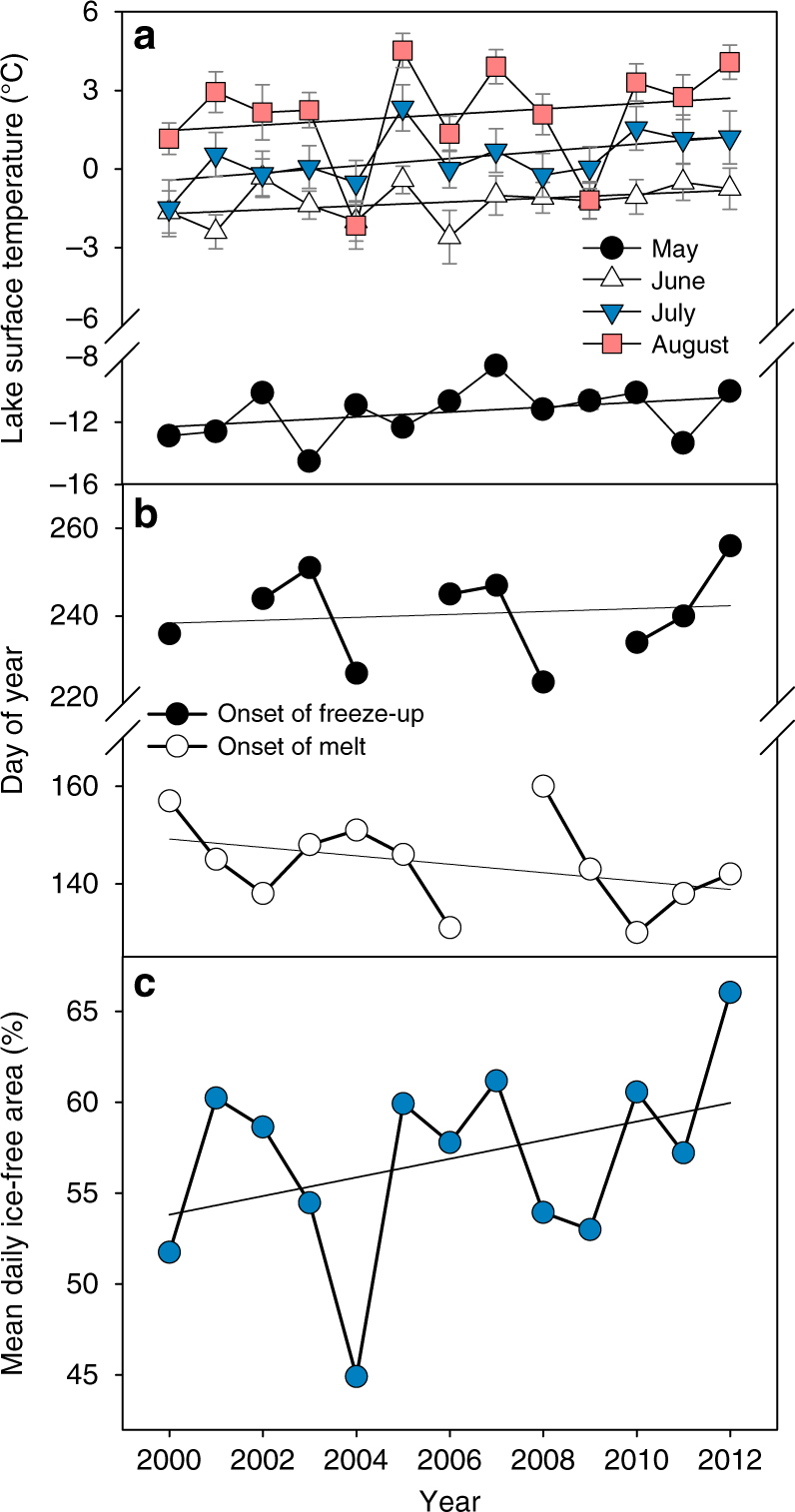
Temporal trends in surface temperature, ice phenology and ice cover at Lake Hazen. **a** Monthly mean (±SE) lake surface temperatures (°C) measured at 30 sites on Lake Hazen. **b** Onset dates (day of year) of melt and freeze-up. **c** Mean daily ice-free area (% of total lake area) on Lake Hazen between May 5 and September 5. For illustrative purposes, linear trend lines are shown, though none are significant (*p* > 0.05)

The Lake Hazen watershed has warmed intensely since the turn of the century. Mean (±SD) summer (June, July, August (JJA)) land surface temperatures of glacier-covered regions of the Lake Hazen watershed increased by 0.21 ± 0.05 °C y^−1^ from 2000 to 2012, representing a 2.6 °C warming over that time period (Fig. 1). Most of this warming occurred from 2007 to 2012 when summer temperatures were 0.9 °C warmer than the period mean (−4.9 °C). Between 1994 and 2010, temperatures in the upper 1 m of soils on the desert landscape near the Lake Hazen base camp rose by 0.14 ± 0.11 °C y^−1^ (Fig. 2), with most warming occurring from 2007 onward. Soil warming was most pronounced in spring. May–June soil temperatures were 4 ± 1 °C warmer in 2007–2012, relative to 1994–2006, likely due to warmer near-surface air temperatures (Fig. 2) driving an earlier onset of snowmelt. Furthermore, the number of days during which soil temperatures were above freezing at 50 cm depth increased by 24 days in July and 22 days in August (Fig. 2), resulting in permafrost soils that, prior to 2007, were frozen year-round now being part of the seasonally thawed active layer for most of the summer, a trend recorded across most northern permafrost sites^16^. Between 2000 and 2012, the monthly mean lake surface temperatures for May (snow-covered ice), June (bare ice following snowmelt), July (moating and breaking up of ice) and August (up to full absence of ice) increased by, on average, 0.16, 0.07, 0.14 and 0.10 °C y^−1^ (Fig. 3), which is significantly greater than the increase in summer surface temperature observed for other seasonally ice-covered lakes around the world (median = 0.048 °C y^−1^; ref. ^17^). More rapid warming in spring advanced the onset of ice break-up by an average of 0.9 d y^−1^, while less intense warming in August delayed freeze-up by 0.3 d y^−1^. On average, the mean ice-free area (5 May to 5 September) of the lake increased by 3 km^2^ y^−1^ (or 0.5% y^−1^) after 2000. Annual daily ice-free area (%) was significantly related to annual August lake surface temperatures (Pearson's correlation *r* = 0.867, *p* = 0.0001). However, annual daily ice-free area (%) was not related to annual glacier runoff volume (see below) (Pearson's correlation *r* = 0.023, *p* = 0.94), suggesting that thermal inertia induced by inputs of relatively warm glacial meltwaters from the watershed in JA (e.g., mean (±SD) 1997–2012 water temperatures in the Abbé, Very and Turnabout rivers were 5.3 ± 1.2, 9.3 ± 1.8 and 9.3 ± 2.3 °C; Environment and Climate Change Canada) was not the primary driver of lake surface warming in a given year. Full ice-off on Lake Hazen became more frequent in recent decades as the lake went ice free for periods of a month or more in 60, 80 and 88% of the years, between 1985 and 1995, 1996 and 2005 and 2006 and 2012, resulting in the progressive loss of multiyear ice (this study and refs.^18,19^).

### Hydrological changes in the Lake Hazen watershed

**Fig. 4:**
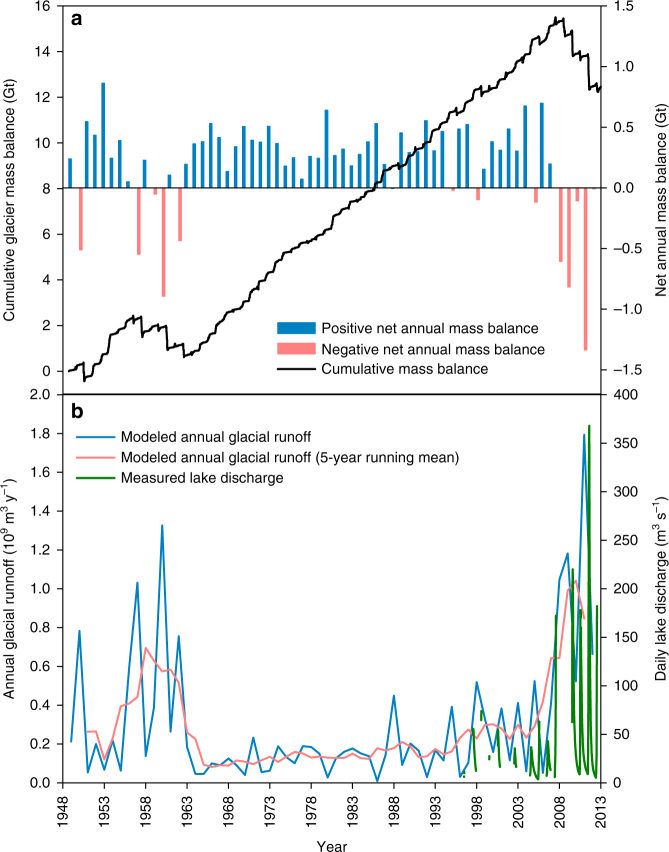
Changes in glacier mass balance, glacial runoff and Lake Hazen discharge. **a** Modeled net annual mass balance (bars) and cumulative mass balance (line) for glaciers in the Lake Hazen watershed for 1948–2012. All values are in Gt, but note that the annual and cumulative mass balances are plotted on different *y*-axis scales. **b** Modeled glacial runoff (annual and 5-year running mean, 1948–2012) compared with measured daily discharge from Lake Hazen at the outflow (Ruggles River) from 1996 to 2012

The hydrological regime of Lake Hazen is primarily, and strongly, controlled by the glaciers within its catchment. Modeled annual glacier mass balances (annual accumulation minus annual ablation) for the period 1949–2012 showed a distinct shift from net mass gain to net mass loss beginning in 2007–2008 (Fig. 4). This change was coincident with rising air temperatures^20,21^ and amplified by positive temperature–albedo feedbacks (whereby higher surface temperatures drive albedo declines that enhance surface warming and/or melt, leading to additional reductions in surface albedo) that together increased both the duration and intensity of meltwater production in summer. This warming caused intense melting events within a short 6–8-week period in June-August, even in the interior high-elevation regions of the glaciers where melt previously occurred infrequently^22^. Modeled mean rates of annual glacier runoff reached 660 kg m^−2^ or 1.8 km^3^ y^−1^ over the entire Lake Hazen catchment in 2011 (Fig. 4). Modeled runoff rates agree well with direct measurements of river discharge at the Lake Hazen outflow (Ruggles River; Fig. 4, Supplementary Fig. 1). For the period 2007–2012 relative to 1996–2006, increased glacier runoff raised water levels in Lake Hazen by 0.8 m on average (Supplementary Fig. 2), and increased mean annual discharge from its outflow by 370%, from 0.49 to 1.8 km^3^. Using modeled glacier runoff values to extend the time series provided by the instrumental record from the Ruggles River, we find that the last time runoff rates were comparable to those in 2007–2012 was during a brief warm period in the 1950s (Fig. 4) that was recorded by the few weather stations operating in the High Arctic during that time^23^ and which is evident in the earliest records of glacier mass balance from this region^24^. Annual runoff in excess of 1 × 10^9^ m^3^ occurred in three of the five years between 2007 and 2012, but only twice in the previous 58 years. The large input of glacier meltwaters into Lake Hazen has reduced the residence time of water in the lake from its historical average of ~89 years^10^ to only 25 years.

### Recent changes informed by the paleolimnological record

**Fig. 5:**
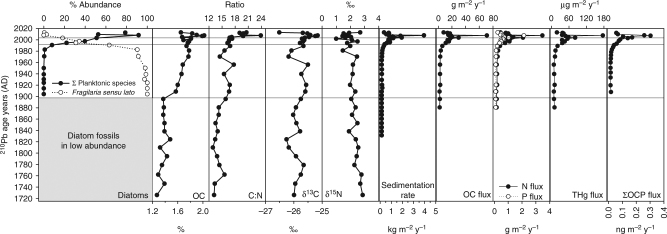
Sediment record of diatom abundance, geochemical parameters, contaminants and sedimentation rates. Diatom, geochemical and contaminant analyses were completed on three separate sediment cores collected in close proximity in May 2013 from the deepest location in Lake Hazen^10^. One of these cores was used for ^210^Pb radiometric dating (also see Supplementary Fig. 7) and calculation of sedimentation rates. This same core was analyzed for organic matter geochemistry and multi-element concentrations. OC organic carbon, C carbon, N nitrogen, P phosphorus, THg total mercury, OCP organochlorine pesticides. See Supplementary Fig. 4 for sediment concentration profiles of N, P and contaminants. The horizontal lines demarcate when diatoms first appear in the paleolimnological record in significant numbers (bottom), when the relative abundance of planktonic diatom species first began to increase (middle) and then surpassed that of benthic species (top)

We used a multi-proxy paleolimnological approach to place the recent warming and its impacts on Lake Hazen within a longer-term context. Analyses of lacustrine sediments (Fig. 5) show that Lake Hazen has recently undergone a major regime shift in response to climate warming. The magnitude of the impacts is unprecedented and exceeds anything observed in the past 300 years, including during the period of warming at the end of the Little Ice Age. Sediment accumulation rates since 2007 (4.2 kg m^−2^ y^−1^) are on average 8 times higher relative to the pre-1948 baseline period (0. 5 kg m^−2^ y^−1^) (Fig. 5), mirroring recent trends in glacial runoff (Fig. 4), which is the main driver of sediment delivery to the lake. Elevated discharge of glacier-fed rivers into the lake has resulted in dense, oxygen-rich turbid underflows^25^ facilitating mixing and oxygenation of bottom waters (Supplementary Fig. 3). This recent summertime ventilation of anoxic bottom waters likely marks a departure from the stable low redox conditions inferred to be historically prevalent at the bottom of the lake when glacial runoff and sedimentation rates were low. Increased sediment delivery has also resulted in increased sequestration of anthropogenic contaminants, such as mercury (Hg) and legacy organochlorine pesticides (OCPs), into lake sediments (Fig. 5). Rising concentrations of legacy OCPs in sediments post 2000 (Supplementary Fig. 4), after a decline from maxima in the 1980s, reflect remobilization of OCPs previously deposited and stored in glaciers^26^, increasing exposure of arctic aquatic foodwebs to legacy contaminants.

Lake Hazen sediments contain low concentrations of organic carbon (OC) (<2.5%; Fig. 5). However, recent increases in sediment inputs have resulted in a parallel 1000% increase in OC accumulation rates (Fig. 5), which is much higher than the 50% increase, on average, in OC accumulation that North American boreal lakes have experienced since 1950^27^. Recent (2007–2012) accumulation rates in Lake Hazen (14–71 g OC m^−2^ y^−1^) are now similar to or greatly exceed those found in boreal and northern temperate lakes (15 ± 9.4 g OC m^−2^ y^−1^)^27^. Profiles of δ^13^C, δ^15^N and C:N ratios in bulk sediments are consistent with gradual organic matter (OM) diagenesis^28^, and show that the source of OM accumulating in Lake Hazen sediments is, and always has been, primarily terrestrially derived. Likely sources of terrestrial OM in this low-productivity High Arctic ecosystem^29^ include vegetation and soils destabilized by increased flow in glacial river channels and deltas, and vegetation and old soil OM previously overrun by glacier advances following the warmer hypsithermal period 9000 to 5000 years bp. Sedimentary profiles of δ^13^C and δ^15^N have been used to evaluate changes in autochthonous algal productivity^28^; however, recent changes in Lake Hazen productivity cannot be assessed in this manner. The δ^13^C and δ^15^N values of contemporary particulate OM in the upper water column (−29.4 and 4.1‰), where productivity is highest, differed from those of older, pre-2000 sedimentary OM (−25.9 and 2.4‰). Intermediate δ^13^C and δ^15^N values (−26.5 and 2.7‰) were observed only in very surface sediments (Fig. 5). Together, these data demonstrate that autochthonous OC in this ultra-oligotrophic lake is rapidly decomposed at the sediment–water interface, rather than accumulated. In fact, rates of decomposition have likely increased with recent increases in OC inputs and summer bottom water oxygenation (Supplementary Fig. 3). Therefore, the sediment archive is not particularly sensitive to changes in algal productivity, which may already be increasing in response to decreased ice cover and increased nitrogen and phosphorus inputs from glacial rivers (Fig. 5).

### Ecological shifts in Lake Hazen

To determine how the ecology of primary producers in the lake has changed as a result of climate-driven changes to the Lake Hazen watershed, algal (diatom) community assemblages were reconstructed from microfossil counts in dated sediments^30^. Prior to ~1890, diatom fossils, although well preserved, were rare (Fig. 5, Supplementary Fig. 5), indicating that algal growth was severely restricted by extensive ice cover on Lake Hazen^31–33^. Subsequently, when temperatures began to rise ca. 1890 as indicated by the ice core record from the Canadian Arctic^34^, taxa common to nearshore habitats flourished^31–33^, specifically *Fragilaria sensu lato* species such as *Staurosirella pinnata, Staurosira construens* and *Staurosira venter* (Supplementary Fig. 5), suggesting development of greater ice-free areas along the lake’s shoreline. The most recent large-scale ecological reorganization began in the late 1980s when planktonic *Cyclotella sensu lato*^35^ increased in relative abundance and eventually supplanted benthic species ca. 1998 (an exceptionally warm summer in the Canadian Arctic^36^) as the dominant taxa in the diatom community (Fig. 5). This reorganization was likely driven in large part by the observed earlier onset of ice break-up, an increase in the growing season ice-free area of the lake and Lake Hazen becoming mostly ice free in late summer, all of which enhanced light penetration in the pelagic environment.

**Fig. 6:**
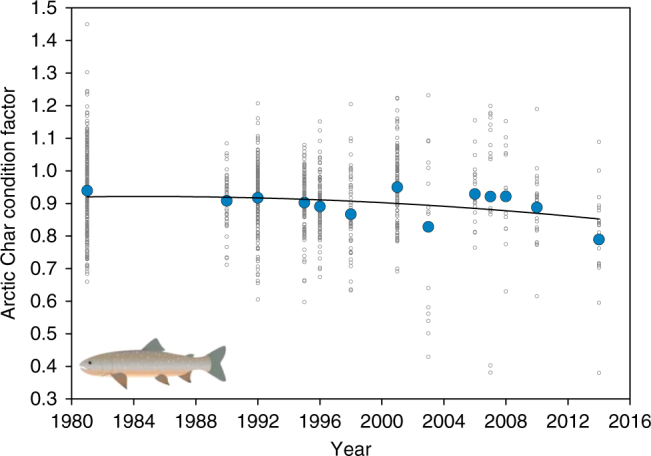
Physiological condition of Arctic Char (*Salvelinus alpinus*) in Lake Hazen. Fulton’s condition factor was calculated for Arctic Char with a mass of 200 g or greater collected between 1981 and 2014. Small open circles are condition factors for individual Arctic Char, whereas larger blue circles are mean condition factors for a given year. A quadratic trend line was fitted to all the data (*p* < 0.001). Arctic Char image credit: Kativik Ilisarniliriniq

Climate-related changes are also impacting the only fish species in Lake Hazen, Arctic Char, the physiological condition of which has declined significantly in recent years (Fig. 6). Although assessing the exact causes of this decline is beyond the scope of this study, one contributing factor may be increased turbidity in the lake, arising from increased discharge of sediment-rich glacier-fed rivers, which impacts the feeding efficiency of this visual predator whose main prey are chironomid midges and young Arctic Char (cannibalism)^37^. As climate warming continues in the region, resulting in accelerated net glacial mass loss and increasing glacier-fed river discharge, we predict that the physiological condition of Arctic Char will progressively decline further. Any decline in the physiological condition in these long-lived, slow-growing fish could threaten what was already thought to be an ecologically sensitive population^9^ of one of the Arctic’s most economically and culturally important species.

In conclusion, the current biogeochemical, limnological and ecological conditions in Lake Hazen have no precedent within the last ~300 years. Although other lakes around the world may respond differently to a warming climate^38^, we show that, because of tight coupling with the cryosphere, changes to the Lake Hazen ecosystem were mediated primarily by increasing glacial melt and loss of lake ice cover. Rising inputs of glacial runoff into Lake Hazen altered the lake’s hydrology and increased the delivery of sediment, OC, nutrients and contaminants, likely enhancing in-lake processes such as net ecosystem productivity and contaminant bioaccumulation. A decrease in seasonal ice cover resulted in warming of surface waters and, more importantly, allowed planktonic algae to fill a niche which was previously climatically inaccessible, re-organizing the ecology of the lake at the base of the foodweb. Collectively, rising air temperatures, increasing glacial melt and runoff, decreasing summer lake ice cover, shifts in primary producer communities and declining fish condition demonstrate the coupling between watershed changes and in-lake conditions and processes. This vast, deep lake, the High Arctic’s largest freshwater ecosystem, has experienced drastic changes in the last decade, despite its volume, thermal inertia and hypothesized resilience to climate change. Such changes, and their consequences, are certain to increase further as warming of northern latitudes continues into the future, undoubtedly jeopardizing the security of traditional freshwater foods and other ecosystem services for northern Indigenous peoples throughout the Arctic.

## Methods

### Land surface temperature and lake ice phenology

Land surface temperatures (LST) and lake ice phenology for 2000–2012 were determined from 8-day Global LST and Emissivity (MOD11A2) and daily Snow Cover (MOD10A1) data from the Moderate Resolution Imaging Spectroradiometer (MODIS) (lpdaac.usgs.gov), respectively. Mean summer (JJA) LST was computed for glaciated surfaces within the watershed (1–2 June to 28–29 August) following Mortimer et al.^22^. MOD11A2 with an LST uncertainty of >2 °C were removed, while remaining pixels with a temperature of >0 °C were assigned a temperature of 0 °C^39^. For each year, mean JJA LSTs were computed for pixels having at least 7 of 12 possible 8-day observations and a linear regression was performed on each pixel having mean JJA LST observations in all 12 years. Ice phenology on Lake Hazen was tracked from 5 May to 5 September, after which cloud cover frequently interfered with MODIS data acquisition. Major phenology events in the lake ice record (onset and end of break-up and freeze-up) were identified at the inflection points in MOD10A1 data. Days having >15% cloud cover were excluded from the analysis and data were interpolated for such periods. Mean daily ice-free area (% of total lake area) on Lake Hazen was calculated each year for the period between 5 May and 5 September. Monthly (MJJA) lake surface temperatures for the period 2000–2012 were quantified for thirty 9 km^2^ areas on Lake Hazen’s surface (approximately 50% of the offshore lake surface area, avoiding contamination from terrestrial landscapes) using MOD11A2 and MYD11A2 subsets (http://daac.ornl.gov/MODIS/).

### Glacier mass balance modeling

A mass budget model that determines glacier surface melt using the temperature-index method was used to estimate glacial runoff volume into Lake Hazen. The model was forced with downscaled and bias-corrected temperature and precipitation fields from the National Centers for Environmental Prediction/National Center for Atmospheric Research Reanalysis. The model and its validations are detailed in the Supplementary Methods of Gardner et al.^20^, with the exception that for this study it was configured to provide output at a daily resolution. Results presented here are the average of three model realizations that produce results that closely match the average of the 1000 model realizations reported in Gardner et al.^20^. The model was additionally validated against the only known in situ surface mass balance measurements collected for any glacier (Gilman Glacier) within the Lake Hazen watershed. Model results agree exceptionally well with the 4 years of in situ observations collected between 1958 and 1962^40^ (Supplementary Fig. 6).

### Lake Hazen water levels and outlet discharge

Lake Hazen water levels and discharge rates at the Ruggles River lake outlet were obtained from the Water Survey Canada (Environment and Climate Change Canada (ECCC)) archived hydrometric data website (wateroffice.ec.gc.ca; Station: 10VK001, Ruggles River at outlet Lake Hazen) for the period 1996–2012.

### Soil temperatures

From 1994 to 2010, soil temperatures were recorded every 6 h at depths of 0 (shielded air temperature at 1 m above the soil surface), 2.5, 5, 10, 20, 35, 50 and 100 cm at a single desert soil site near the Lake Hazen base camp using YSI thermistors and a XL-800 datalogger. Thermistors were attached to a 2.8 cm diameter plastic tube inserted into an augured hole to minimize disturbance. Daily and monthly means were quantified from raw data at all depths for trend analyses and spatio-temporal heat mapping. Mean annual changes in monthly soil temperature at all depths during the measurement record were determined using slopes from Marquardt–Levenberg linear curve fittings (SigmaPlot, Systat Software Inc., v10). Differences in mean monthly soil temperatures before and after 2007 were determined to map changes in soil heating between the periods. During years when full monthly records existed for each depth (12–14 of 17 years), we enumerated the number of days each month when mean daily soil temperatures were above freezing (0 °C). We then quantified the mean difference in the number of days above freezing between the pre- and post-2007 period.

### Sediment collection and field processing

Three intact sediment cores with clearly defined sediment–water interfaces were collected in close proximity to one another in May 2013 from the location of maximum depth (260 m) in Lake Hazen^10^ using a UWITEC gravity corer with an 8.6 cm inner diameter PVC tube. Cores were carefully returned to the Lake Hazen field laboratory where they were extruded and sectioned into 0.5 cm intervals on the same day. Each section was placed into a polypropylene screw capped jar, frozen immediately on-site in a propane-powered freezer and kept frozen until analysis.

### Sediment dating

One of the sediment cores was dated using the ^210^Pb Constant Rate of Supply (CRS) age model^41–43^ (Supplementary Fig. 7). The ^210^Pb and ^226^Ra activities for the core were quantified using gamma-ray spectrometry (ECCC, Burlington, Ontario). The ^137^Cs, released by above ground nuclear weapons testing, with peak ^137^Cs fallout ca. 1963, were analyzed using gamma-ray spectrometry. To improve the ^210^Pb chronology accuracy, total unsupported ^210^Pb inventories were constrained with the depth of peak ^137^Cs activity referenced to 1963 (see ref. ^41^; Eqs. (35) and (36)). Sediment ages below the deepest ^210^Pb dating horizon were extrapolated from the relation between sediment cumulative dry mass and the CRS-determined ages in the dated portion of the core, excluding the recent post-2000 period of high sedimentation rates. Sediment dry mass accumulation rates and fluxes of its constituents were normalized for sediment focusing as described elsewhere^44–46^ using the unsupported ^210^Pb inventory previously measured in soil near Lake Hazen^44^ as the local value for direct atmospheric ^210^Pb fallout.

### Sediment multi-element, isotope and contaminant analyses

Sediment samples were analyzed for concentrations of multi-elements at the ECCC National Laboratory for Environmental Testing (Burlington, Ontario). Freeze-dried sediments were first digested in a solution of 9:2:1 HNO_3_:HCl:H_2_O_2_ under pressure and high temperature (200 °C) in a microwave oven. The residue was then further digested with 4:1 HF:HNO_3_ at 90 °C. The digest was reconstituted to the original solution ratio of 9:2 HNO_3_:HCl and diluted to a fixed volume of 100 mL with reagent water for analysis by inductively coupled argon plasma-collision/reaction cell mass spectrophotometry (CRC-ICP-MS) using discrete sampling pneumatic nebulization. Standard Reference Materials (SRMs) NRCC MESS and NIST RM 8704 analyzed with samples were consistently within ± 7% of certified values.

Freeze-dried sediments, washed with dilute HCl to remove carbonate, were analyzed for δ^13^C, δ^15^N, %N and %C with a Delta Plus (Thermo) continuous flow isotope ratio mass spectrometer coupled to a Carlo Erba 1500 Elemental Analyzer in the Environmental Geochemistry and Environmental Isotope laboratories, University of Waterloo. The δ^15^N and δ^13^C ratios (‰) were determined using the equation δ*R*‰ = ((*R*_sample_ = *R*_standard_) − 1) × 1000, where *R*_sample_ was the ratio of ^15^N/^14^N or ^13^C/^12^C in the sample, and *R*_standard_ for ^13^C or ^15^N was referenced to that in Vienna Pee Dee Belemnite and atmospheric air, respectively. Precision of analysis was 0.2 and 0.3‰ for δ^13^C and δ^15^N, and 0.3% and 0.03% for %C and %N.

Freeze-dried sediments were analyzed for total mercury (THg) concentrations using a Milestone DMA-80 direct mercury analyzer following USEPA Method 7473 at the ECCC National Laboratory for Environmental Testing. SRMs NRCC MESS-3 and SRM-2976 analyzed with samples were within ±5% of certified values.

Organochlorine pesticides and related compounds (OCPs) were determined by ALS Environmental, Burlington, ON (www.alsglobal.com) in select sediment sample intervals using USEPA Method 1699. In brief, wet sediments were mixed with anhydrous Na_2_SO_4_ to give a free-flowing mixture. Percent moisture was determined separately on samples analyzed for multi-elements to allow for results to be expressed on a dry weight basis. Laboratory blanks consisting of all lab reagents were run with each batch of 12 samples. The ^13^C_12_-PCB-133 was added prior to extraction as an internal standard. The Na_2_SO_4_ solid mixture was Soxhlet extracted overnight with dichloromethane. The extract was spiked with a suite of ^13^C-labeled OCPs prior to instrumental analysis for target quantification. The raw extracts were cleaned by gel permeation chromatography to remove pigment co-extractives and then subjected to a silica gel column cleanup. The OCPs were analyzed by gas chromatography-high-resolution mass spectrometry (mass resolution >8000) and quantified relative to ^13^C-OCPs via isotope dilution. All data were recovery corrected for extraction losses relative to ^13^C_12_-PCB-133 responses. Sediment SRM NIST 1941b was analyzed with each batch of 12 samples. Results for certified OCP analytes were within ±20% of certified values. The OPCs reported here include alpha-, beta- and gamma-HCH, heptachlor, aldrin heptachlor epoxide B, oxychlordane *trans*- and* cis*-chlordane, *trans*-nonachlor, dieldrin, endrin *cis*-nonachlor, alpha- and beta-endosulfan, endosulfan sulfate, 2,4′- and 4,4′-DDE, 2,4′- and 4,4′-DDD, 24′- and 4,4′-DDT, methoxychlor, mirex, and Parlar-26, -50 and -62.

### Siliceous microfossil identification and abundance counts

Preparation of sediments for siliceous microfossil identification and enumeration followed standard procedures^30^. At minimum, 200 microfossil valves were counted for each sediment interval, although the bottom two intervals counted contained low abundances such that only ~100 valves could be identified and enumerated. However, given the low diversity of microfossils in the lower sediment intervals, these numbers provided reliable counts.

### Arctic Char condition factor

Lake Hazen Arctic Char condition was calculated from morphometric measures made on Arctic Char collected in 1981, 1990, 1992, 1995, 1996, 1998 and 2001^47^, as well as on Arctic Char subsequently collected in 2003 (*N* = 17), 2006 (*N* = 22), 2007 (*N* = 18), 2008 (*N* = 15), 2010 (*N* = 25) and 2014 (*N* = 19)^48–50^. Fulton’s condition factor (CF = 100 × weight [g]/fork length^3^ [cm^3^])^51^ was calculated for all Arctic Char >200 g.

### Data availability

The data that support the findings of this study are all available from the corresponding author upon reasonable request.

## Electronic supplementary material

Supplementary Information(PDF 10916 kb)

## References

[1] Serreze MC, Barry RG (2011). Processes and impacts of Arctic amplification: a research synthesis. Glob. Planet. Change.

[2] Taylor KE, Stouffer RJ, Meehl GA (2012). An overview of CMIP5 and the experiment design. Bull. Am. Meteorol. Soc..

[3] van Oldenborgh, G. J.* KNMI Climate Explorer* (Koninklijk Netherlands Meteorologisch Institut (KNMI), De Bilt, The Netherlands, 1999).

[4] Prowse T (2015). Arctic freshwater synthesis: summary of key emerging issues. J. J. Geophys. Res. Biogeosci..

[5] Laudon H (2017). Save northern high-latitude catchments. Nat. Geosci..

[6] Herdendorf CE (1982). Large Lakes of the World. J. Gt. Lakes. Res..

[7] Nuttall M (2005). Encyclopedia of the Arctic.

[8] McGhee, R. *The Last Imaginary Place: A Human History of the Arctic World* (Oxford University Press, Oxford, New York, 2005).

[9] Babaluk JA (1997). Evidence for non-anadromous behaviour of arctic charr (Salvelinus alpinus) from Lake Hazen, Ellesmere Island, northwest Territories, Canada, based on scanning proton microprobe analysis of otolith strontium distribution. Arctic.

[10] Köck G (2012). Bathymetry and sediment geochemistry of Lake Hazen (Quttinirpaaq National Park, Ellesmere Island, Nunavut). Arctic.

[11] Emmerton CA (2016). The importance of freshwater systems to the net atmospheric exchange of carbon dioxide and methane with a rapidly changing high Arctic watershed. Biogeosciences.

[12] Wrona FJ (2016). Transitions in Arctic ecosystems: ecological implications of a changing hydrological regime. J. Geophys. Res. Biogeosci..

[13] Chen G (2014). Proximity to ice fields and lake depth as modulators of paleoclimate records: a regional study from southwest Yukon, Canada. J. Paleolimnol..

[14] Smol JP, Douglas MSV (2007). Crossing the final ecological threshold in high Arctic ponds. Proc. Natl. Acad. Sci. USA.

[15] Smol JP (2005). Climate-driven regime shifts in the biological communities of arctic lakes. Proc. Natl. Acad. Sci. USA.

[16] Stocker, T. F. et al. *IPCC 2013: Climate Change 2013: The Physical Science Basis. Contribution of Working Group I to the Fifth Assessment Report of the Intergovernmental Panel on Climate Change*, 1535 (Cambridge University Press, Cambridge, New York, 2013).

[17] O’Reilly CM (2015). Rapid and highly variable warming of lake surface waters around the globe. Geophys. Res. Lett..

[18] Latifovic R, Pouliot D (2007). Analysis of climate change impacts on lake ice phenology in Canada using the historical satellite data record. Remote Sens. Environ..

[19] Surdu CM, Duguay CR, Prieto DF (2016). Evidence of recent changes in the ice regime of lakes in the Canadian High Arctic from spaceborne satellite observations. Cryosphere.

[20] Gardner AS (2011). Sharply increased mass loss from glaciers and ice caps in the Canadian Arctic Archipelago. Nature.

[21] Sharp M (2011). Extreme melt on Canada’s Arctic ice caps in the 21st century. Geophys. Res. Lett..

[22] Mortimer CA, Sharp M, Wouters B (2016). Glacier surface temperatures in the Canadian High Arctic, 2000–15. J. Glaciol..

[23] Sharp, M. et al. in *Global Land Ice Measurements from Space* (eds Kargel, J. S. et al.) 205–228 (Springer, Berlin Heidelberg, 2014).

[24] Koerner, R. M. in *Satellite Image Atlas of Glaciers of the World* (eds Williams, R. S. & Ferrigno, J. G.) J111–J146 (U.S. Geological Survey, 2002).

[25] Crookshanks S, Gilbert R (2008). Continuous, diurnally fluctuating turbidity currents in Kluane Lake, Yukon Territory. Can. J. Earth Sci..

[26] Bogdal C (2009). Blast from the past: melting glaciers as a relevant source for persistent organic pollutants. Environ. Sci. Technol..

[27] Heathcote AJ, Anderson NJ, Prairie YT, Engstrom DR, del Giorgio PA (2015). Large increases in carbon burial in northern lakes during the Anthropocene. Nat. Commun..

[28] Meyers, P. A. & Teranes, J. L. in *Tracking Environmental Change Using Lake Sediments* (eds Last, W. M. & Smol, J. P.) 239–270 (Kluwer Academic Publishers, Boston, 2001).

[29] Emmerton CA (2016). Net ecosystem exchange of CO_2_ with rapidly changing high Arctic landscapes. Glob. Change Biol..

[30] Battarbee, R. W. in *Tracking Environmental Change Using Lake Sediments* (eds Last, W. M. & Smol, J. P.) 155–202 (Kluwer Academic Publishers, Boston, 2001).

[31] Antoniades D (2007). Abrupt environmental change in Canada’s northernmost lake inferred from fossil diatom and pigment stratigraphy. Geophys. Res. Lett..

[32] Michelutti N, Douglas MSV, Smol JP (2003). Diatom response to recent climatic change in a high arctic lake (Char Lake, Cornwallis Island, Nunavut). Global Planet Change.

[33] Perren BB (2012). Twentieth-century warming revives the world’s northernmost lake. Geology.

[34] Fisher D (2012). Recent melt rates of Canadian arctic ice caps are the highest in four millennia.. Global Planet Change.

[35] Rühland KM, Paterson AM, Smol JP (2015). Lake diatom responses to warming: reviewing the evidence. J. Paleolimnol..

[36] Atkinson DE (2006). Canadian cryospheric response to an anomalous warm summer: a synthesis of the climate change action fund project “The state of the arctic cryosphere during the extreme warm summer of 1998”. Atmos. Ocean.

[37] Robertson, M. J., Scruton, D. A., Gregory, R. S. & Clarke, K. D. *Effect of Suspended Sediment on Freshwater Fish and Fish Habitat* (Fisheries and Oceans Canada, 2006).

[38] Adrian R (2009). Lakes as sentinels of climate change. Limnol. Oceanogr..

[39] Hall DK, Williams RS, Luthcke SB, Digirolamo NE (2008). Greenland ice sheet surface temperature, melt and mass loss: 2000–06. J. Glaciol..

[40] Sagar RB (1964). Meteorological and glaciological observations on the Gilman Glacier, northern Ellesmere Island. Geogr. Bull..

[41] Appleby, P. G. in *Tracking Environmental Change Using Lake Sediments* (eds Last, W. M. & Smol, J. P.) 171–203 (Kluwer Academic Publishers, Boston, 2001).

[42] Robbins, J. A. in *The Biogeochemistry of Lead in the Environment* (ed. Nriagu, J. O.) 85–393 (Elsevier, Amsterdam, 1978).

[43] Sanchez-Cabeza JA, Ruiz-Fernandez AC (2012). Pb-210 sediment radiochronology: an integrated formulation and classification of dating models. Geochim. Cosmochim. Acta.

[44] Lockhart WL (1998). Fluxes of mercury to lake sediments in central and northern Canada inferred from dated sediment cores. Biogeochemistry.

[45] Perry E, Norton SA, Kamman NC, Lorey PM, Driscoll CT (2005). Deconstruction of historic mercury accumulation in lake sediments, Northeastern United States. Ecotoxicology.

[46] Muir DCG (2009). Spatial trends and historical deposition of mercury in Eastern and Northern Canada inferred from lake sediment cores. Environ. Sci. Technol..

[47] Babaluk, J. A., Sawatzky, C. D., Wastle, R. J. & Reist, J. D. *Biological Data of Arctic Char*, Salvelinus alpinus*, from Lake Hazen, Quttinirpaaq National Park, Nunavut, 1958–2001* (Fisheries and Oceans Canada, 2007).

[48] Gantner N (2009). Temporal trends of mercury, cesium, selenium, thallium in arctic char (*Salvelinus alpinus*) from Lake Hazen, Nunavut, Canada: effects of trophic position, size, and age. Environ. Toxicol. Chem..

[49] Gantner N (2010). Mercury concentrations in landlocked arctic char (Salvelinus alpinus) from the Canadian Arctic. Part I: insights from trophic relationships in 18 lakes. Environ. Toxicol. Chem..

[50] Muir, D. C. G, Köck, G. & Wang, X. in *Synopsis of Research Conducted under the 2014-2015 Northern Contaminants Program* (ed Aboriginal Affairs and Northern Development Canada) 247–255 (Aboriginal Affairs and Northern Development Canada, Ottawa, 2015).

[51] Bolger T, Connolly PL (1989). The selection of suitable indexes for the measurement and analysis of fish condition. J. Fish. Biol..

