# Supplementary material for: The world’s largest High Arctic lake responds rapidly to climate warming
Source: Nat Commun. 2018 Mar 29;9:1290. doi: 10.1038/s41467-018-03685-z (PMC5876346; doi:10.1038/s41467-018-03685-z)
Supplement: Supplementary file 1 — Supplementary Information(PDF 10916 kb) [file 41467_2018_3685_MOESM1_ESM.pdf]

Supplementary Information (Figures)

The world's largest High Arctic lake succumbs to climate warming

Lehnherr et al.

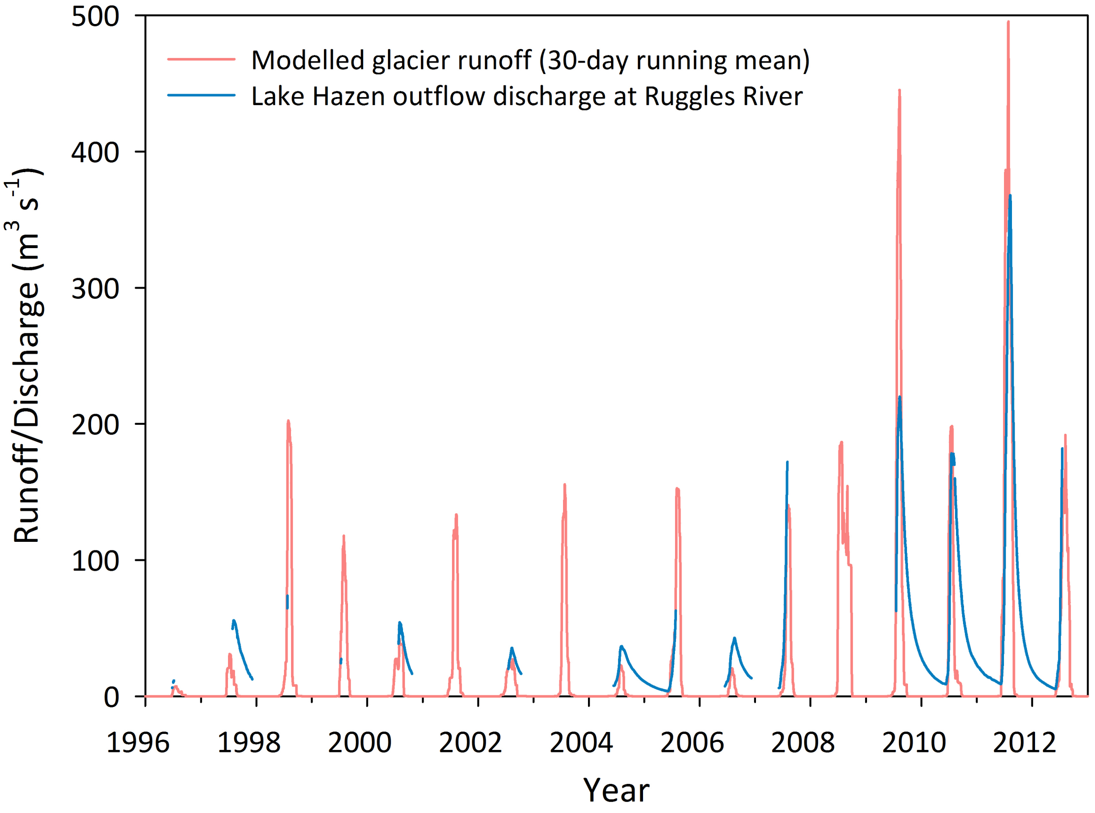

Supplementary Fig. 1 Comparison of modelled glacier runoff and measured Lake Hazen outflow discharge at the Ruggles River for the period 1996-2012**.** Note that each year, modelled glacier runoff into Lake Hazen occurs over a much shorter time period during summer than discharge from the lake due to base flow during winter in the Ruggles River and a storage lag period within Lake Hazen itself.

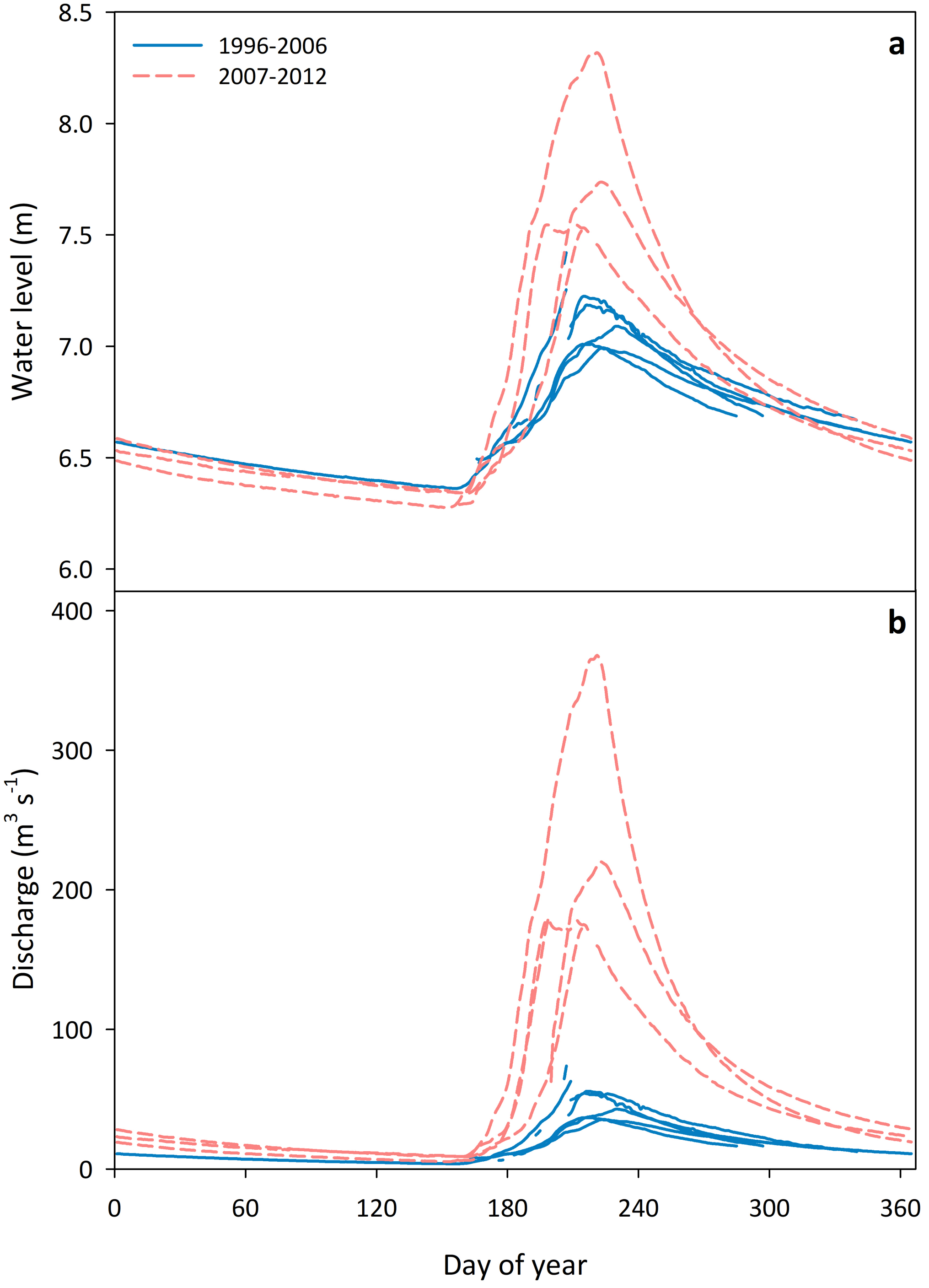

Supplementary Fig. 2 Water **a** level and **b** discharge measured at the Lake Hazen outflow at the Ruggles River for the period 1996-2012. Not all years had complete records due to sensor and instrumentation failure.

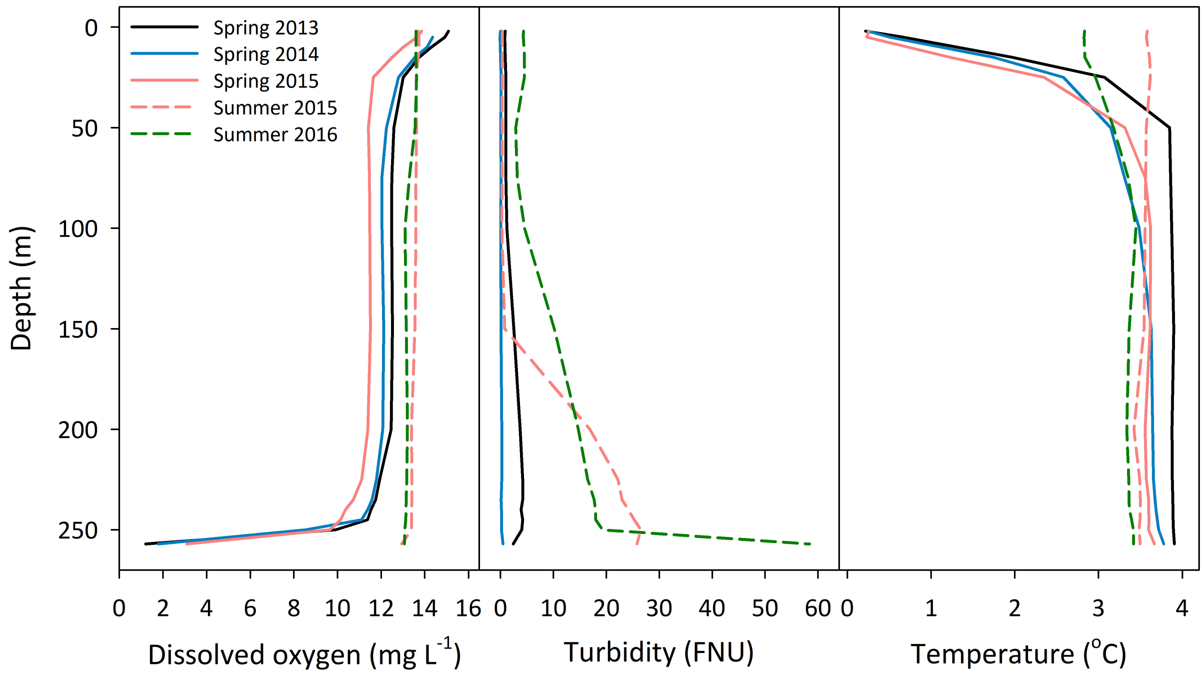

Supplementary Fig. 3 Water column profiles of dissolved oxygen, turbidity and water temperature under the ice (May/June 2013, 2014, 2015) and during the open water period (July/August 2015, 2016) in Lake Hazen. Profiles were measured using an EXO2 sonde equipped with optical dissolved oxygen, turbidity and temperature sensors. A malfunctioning turbidity probe prevented turbidity measures in Spring 2015.

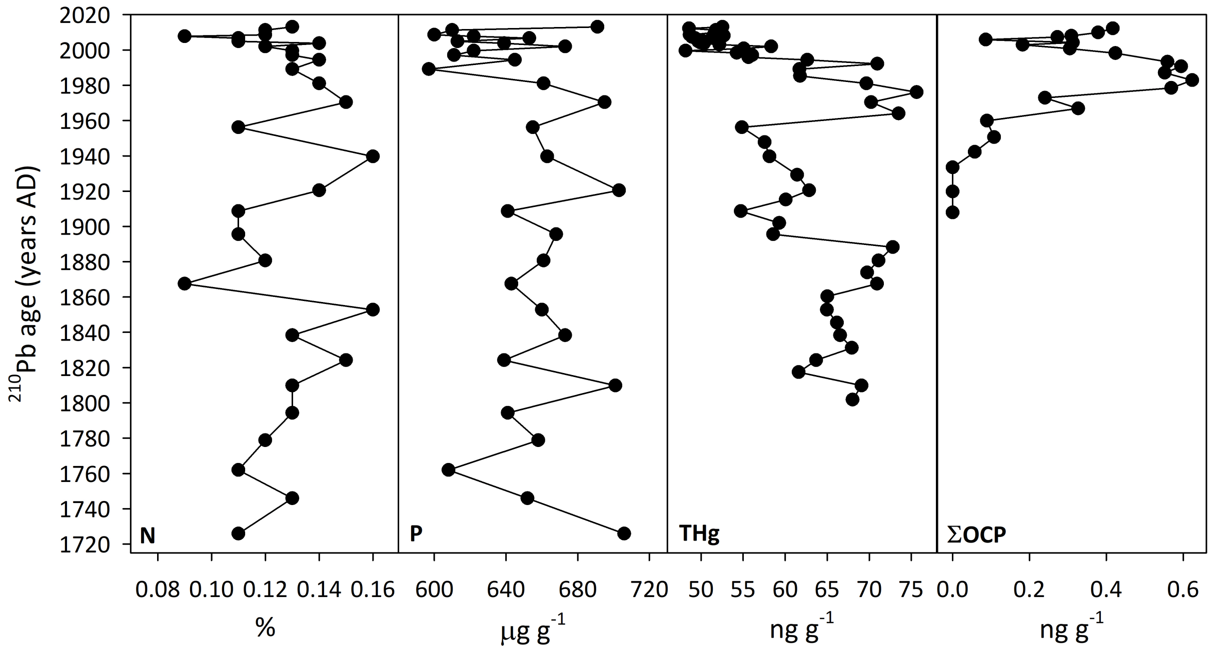

Supplementary Fig. 4 Percent (%) nitrogen (N) and concentrations of phosphorus (P) and contaminants in Lake Hazen sediments. See Fig. 5 for sedimentation rates and fluxes of these parameters over time. THg (total mercury); ΣOCP (sum of organochlorine pesticides).

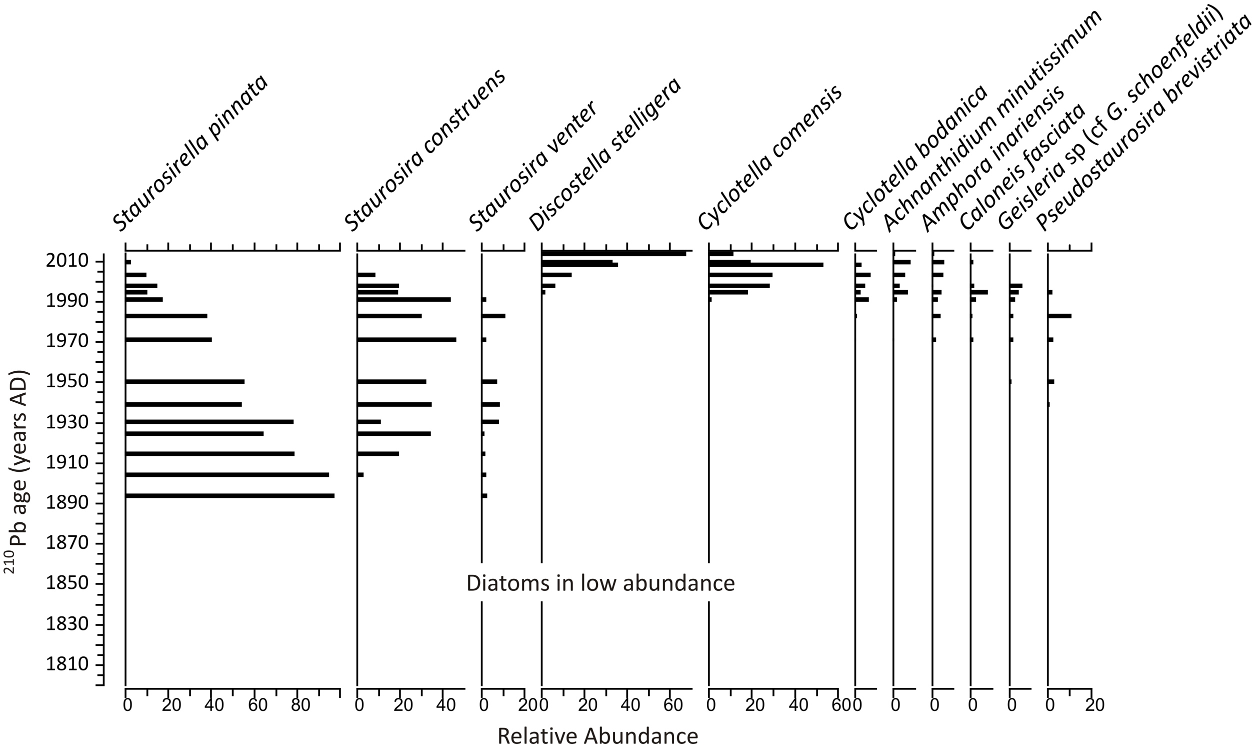

Supplementary Fig. 5 Relative abundances of different siliceous microfossils (diatoms) identified in Lake Hazen sediments. Prior to ~1890, diatom abundance was too low for proper enumeration. The genera *Staurosira* and *Pseudostaurosira* are referred to as *Fragilaria sensu lato* (benthic species), while the genera *Cyclotella* and *Discostella* are together referred to as *Cyclotella sensu lato* (planktonic species).

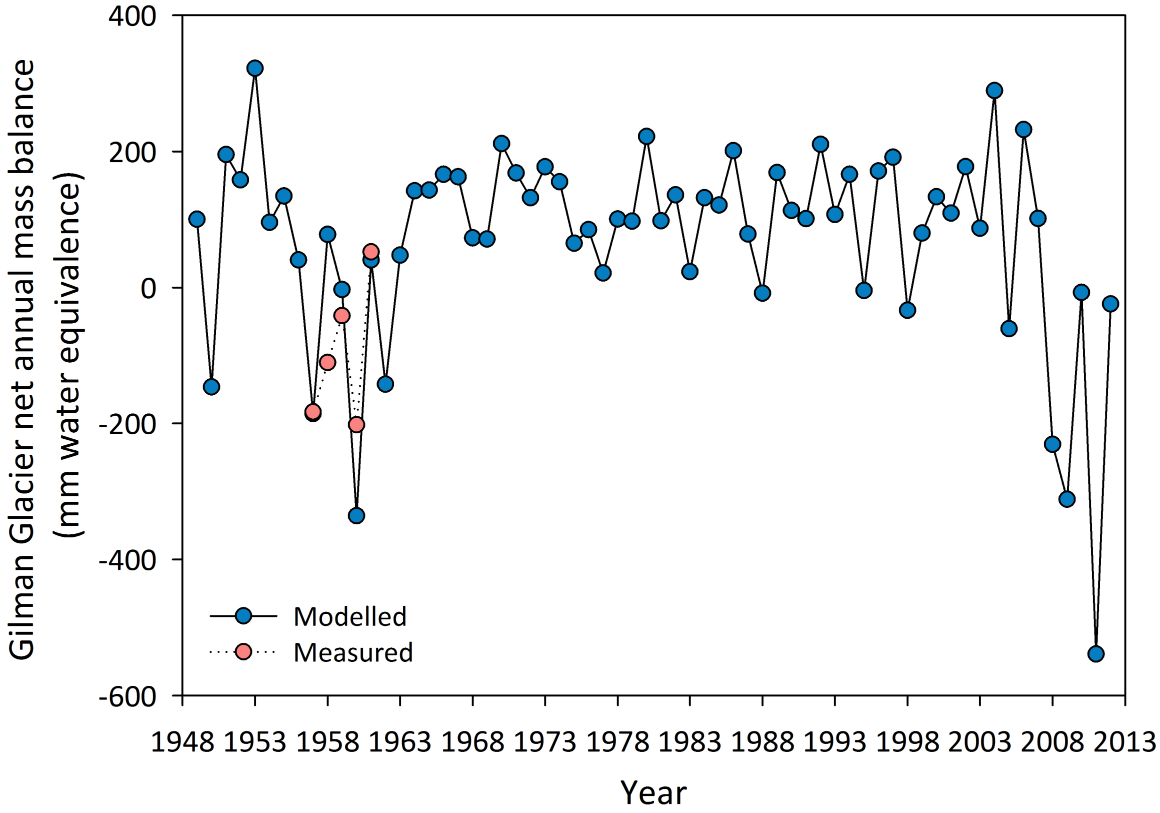

Supplementary Fig. 6 Validation of the glacier mass-balance model. There is good agreement between modelled net annual mass balance values for the Gilman Glacier, located within the Lake Hazen watershed, and empirical measurements made on the same glacier from 1958-1962.[1]

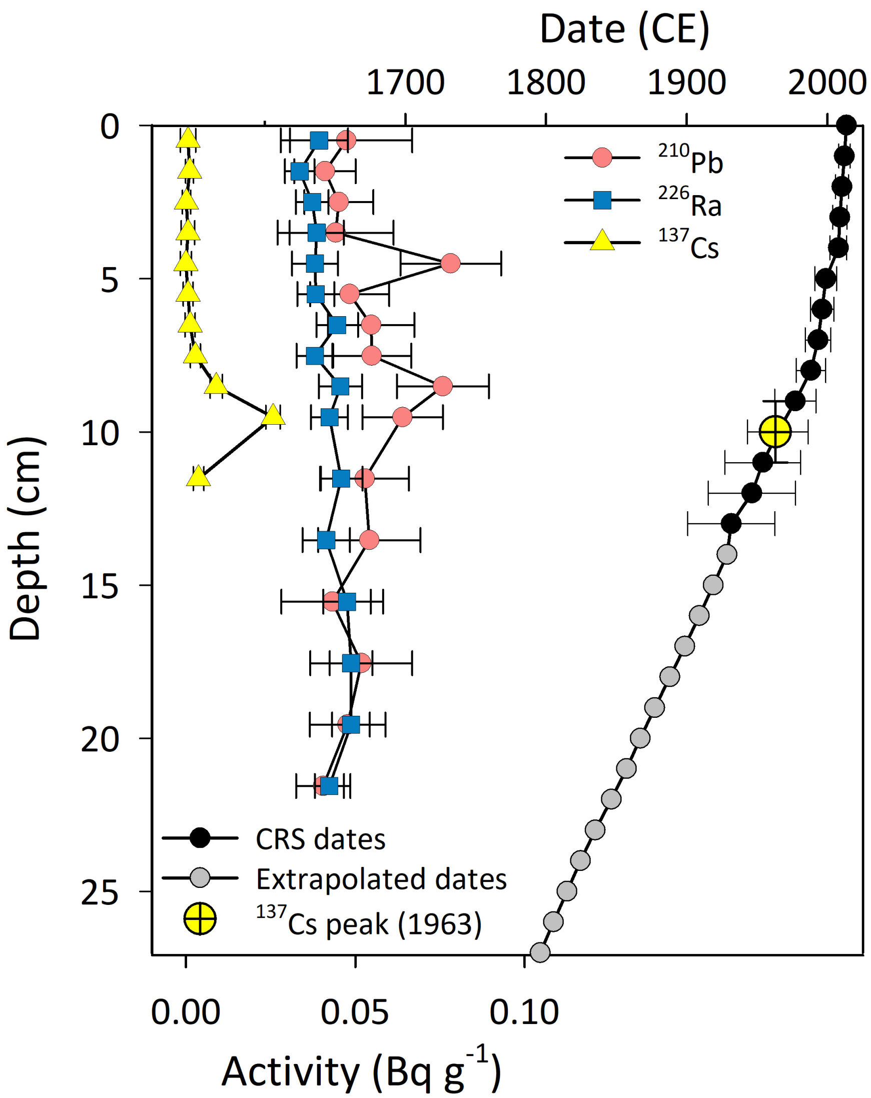

Supplementary Fig. 7 Profiles of ^{210}Pb, ^{226}Ra and ^{137}Cs activity, and the measured Constant Rate of Supply (CRS) and extrapolated dates down core for one of the three Lake Hazen sediment cores collected. In addition to radiometric dating, this core was also analyzed for multi-elements and organic matter geochemistry. Error bars denote ±1 S.D.

References:

1. Sagar, R. B. Meteorological and glaciological observations on the Gilman Glacier, northern Ellesmere Island. *Geograph. Bull.* **22**, 13-56 (1964).
